# Endogenous hormones and risk of invasive breast cancer in pre- and post-menopausal women: findings from the UK Biobank

**DOI:** 10.1038/s41416-021-01392-z

**Published:** 2021-04-16

**Authors:** Sandar Tin Tin, Gillian K. Reeves, Timothy J. Key

**Affiliations:** Cancer Epidemiology Unit, Nuffield Department of Population Health, University of Oxford, Oxford, UK

**Keywords:** Epidemiology, Breast cancer

## Abstract

**Background:**

Some endogenous hormones have been associated with breast cancer risk, but the nature of these relationships is not fully understood.

**Methods:**

UK Biobank was used. Hormone concentrations were measured in serum collected in 2006–2010, and in a repeat subsample (*N* ~ 5000) in 2012–13. Incident cancers were identified through data linkage. Cox regression models were used, and hazard ratios (HRs) corrected for regression dilution bias.

**Results:**

Among 30,565 pre-menopausal and 133,294 post-menopausal women, 527 and 2,997, respectively, were diagnosed with invasive breast cancer during a median follow-up of 7.1 years. Cancer risk was positively associated with testosterone in post-menopausal women (HR per 0.5 nmol/L increment: 1.18; 95% CI: 1.14, 1.23) but not in pre-menopausal women (*p*_heterogeneity_ = 0.03), and with IGF-1 (insulin-like growth factor-1) (HR per 5 nmol/L increment: 1.18; 1.02, 1.35 (pre-menopausal) and 1.07; 1.01, 1.12 (post-menopausal); *p*_heterogeneity_ = 0.2), and inversely associated with SHBG (sex hormone-binding globulin) (HR per 30 nmol/L increment: 0.96; 0.79, 1.15 (pre-menopausal) and 0.89; 0.84, 0.94 (post-menopausal); *p*_heterogeneity_ = 0.4). Oestradiol, assessed only in pre-menopausal women, was not associated with risk, but there were study limitations for this hormone.

**Conclusions:**

This study confirms associations of testosterone, IGF-1 and SHBG with breast cancer risk, with heterogeneity by menopausal status for testosterone.

## Background

Some endogenous hormones have an important role in carcinogenesis, probably by promoting cell proliferation and increasing the chances of random genetic errors.^1^ Breast cancer, the most common cancer in women worldwide,^2^ is known to be hormone-dependent; reproductive and hormonal factors such as early menarche, low parity, late menopause, and in post-menopausal women, hormone replacement therapy and adiposity increase the risk of breast cancer.^3–5^

Previous epidemiological studies have associated circulating sex hormones (oestrogens and androgens) and the growth factor insulin-like growth factor-1 (IGF-1) with an increased risk of breast cancer.^5–8^ The results for sex hormones have been less conclusive in pre-menopausal women due to fewer data being available in this group, and it is not clear if the associations differ by menopausal status. We, therefore, investigated the associations between serum concentrations of testosterone, sex hormone-binding globulin (SHBG) and IGF-1 and the risk of invasive breast cancer in pre- and post-menopausal women in UK Biobank. We also assessed the association with oestradiol, but the data were available for pre-menopausal women only.

With data on hormones for over 30,000 pre-menopausal and over 130,000 post-menopausal women included in this analysis, this is as far as we are aware the largest single study on this topic, and therefore enabled us to undertake a number of important subgroup analyses. Availability of repeat blood samples collected four years after recruitment in about 5000 women also enabled us to account for regression dilution bias (i.e. attenuation of the regression slope due to measurement errors and variations over time in hormone concentrations)^9,10^ and provide robust estimates of the magnitudes of risk per increment in hormone concentrations.

## Methods

### Data source

Data from UK Biobank, a large prospective cohort study, were used (project reference number 3248, approved August 2013). Study design and methodology has been described elsewhere.^11^

### Study participants

UK Biobank involves about 500,000 adults, including over 270,000 women, aged between 40-69 years who were recruited in 22 assessment centres between 2006 and 2010. During the initial assessment centre visit, participants completed a touchscreen questionnaire that included questions on socio-demographics, family history of disease and early life factors, lifestyle and environment, psychosocial factors, health and medical history, and sex-specific factors. Participants also underwent physical measurements, including body size and composition, and provided blood and urine samples. Their health is being followed long-term, principally through electronic linkage to a wide range of health record systems including cancer and death registries.

For the current analyses, women were categorised as pre-menopausal if they were younger than 50 years of age and answered ‘no’ to the question “Have you had your menopause (periods stopped)?”. They were categorised as post-menopausal if they reported “yes” to the above question, or were 55 years or older, or reported a bilateral oophorectomy. Women were excluded from the analyses if they reported being pregnant or currently using oral contraceptive pills (pre-menopausal women), currently using hormone replacement therapy (post-menopausal women), had a prior diagnosis of cancer (except for non-melanoma skin cancer), benign brain tumour or breast carcinoma in situ (identified through linkage to national cancer registries), or had missing data on time since last menstrual period (pre-menopausal women) or concentrations of hormones and SHBG (Supplementary Fig. S1).

### Hormone assays

Blood samples were collected from all participants at the time of recruitment. Repeat blood samples were also collected from a subsample of participants in 2012–13, on average four years after baseline. Hormones and SHBG were measured using chemiluminescent immunoassays in UK Biobank’s purpose-built facility in Stockport, UK. Serum concentrations of testosterone, SHBG and oestradiol (reported here only for pre-menopausal women as almost all post-menopausal women had a very low concentration which was below the reportable range for the assay) were measured on the UniCel DXI 800 (Beckman Coulter, Brea, USA) and the concentration of IGF-1 was measured on the Liaison® XL (DiaSorin, Saluggia, Italy).

If the test result for a given hormone was missing due to a very low serum concentration, the concentration was set at three-quarters of the minimum reportable value (*N* = 25,428 for testosterone (1934 pre-menopausal women and 23,494 post-menopausal women); *N* = 7 for SHBG (2 pre-menopausal women and 5 post-menopausal women); *N* = 3 for IGF-1 (post-menopausal women only); and *N* = 6258 for oestradiol (pre-menopausal women only)). The minimum reportable concentrations were 0.35 nmol/L for testosterone, 0.33 nmol/L for SHBG, 1.30 nmol/L for IGF-1 and 175 pmol/L for oestradiol.^12^ If the test result was missing due to a very high serum concentration, the concentration was set at the maximum reportable value (*N* = 55 for SHBG; 25 pre-menopausal women and 30 post-menopausal women). The maximum reportable concentration of SHBG was 242 nmol/L.^12^ Concentrations of free testosterone and oestradiol were calculated assuming that the binding of these hormones to serum SHBG and albumin follows the law of mass action.^13^ Serum albumin concentration was measured using a dye-binding assay as bromocresol green (BCG) on the AU5800 (Beckman Coulter, Brea, USA).

Repeat measurements were available for testosterone in 5316 women (790 pre-menopausal women and 4526 post-menopausal women); for SHBG in 4646 women (664 pre-menopausal women and 3982 post-menopausal women), for IGF-1 in 5349 women (797 pre-menopausal women and 4552 post-menopausal women) and for oestradiol in 755 pre-menopausal women (note that pre-menopausal women here were those who were categorised as pre-menopausal at baseline; some of them could be peri- or post-menopausal at the time of repeat measurements). There were significant Pearson correlations (*p* < 0.0001) between baseline and repeated measures of testosterone (*r* = 0.58), SHBG (*r* = 0.80) and IGF-1 (*r* = 0.78); for oestradiol, it was not possible to demonstrate correlations over time because few of the repeat samples were collected in the same phase of the menstrual cycle as the baseline sample, and some women became peri- or post-menopausal during follow-up.

### Outcome assessment

Incident cancer cases were identified through linkage to national cancer and death registries. The linked data were available up to 31st March 2016 for England and Wales and 31st October 2015 for Scotland. All registrations coded as C50 using the 10th Revision of the International Classification of Diseases (ICD-10) were considered as invasive breast cancer cases.

### Statistical analysis

Analyses were undertaken separately for pre- and post-menopausal women (at recruitment). STATA 15.1 (StataCorp, College Station, Texas) was used for all analyses.

Cox proportional hazards regression modelling was performed with a diagnosis of invasive breast cancer as the failure variable, and diagnosis of any other cancer (with the exception of non-melanoma skin cancer), loss to follow-up, death or end of follow-up as censored observations. Attained age was used as the time scale. Models were stratified by age at recruitment (in 2-year categories for pre-menopausal women and in 5-year categories for post-menopausal women), quintiles of Townsend deprivation index (measure of material deprivation calculated based on the preceding national census areas) and region of recruitment (Scotland, Wales, North-East England, North-West England, Yorkshire and the Humber, East Midlands, West Midlands, South-West England, South-East England and London). The proportional hazards assumption was checked using Schoenfeld residuals, and no evidence of violation was detected.

Hormone concentrations in pre-menopausal women were standardised for the day of the menstrual cycle with residuals from the mean for each day as described previously.^6^ Median values from the quintiles of hormone concentrations were used to estimate hazard ratios (HRs) and 95% confidence intervals (CIs) on a continuous scale (per 0.5 nmol/L increment for total testosterone, per 10 pmol/L increment for calculated free testosterone, per 30 nmol/L for SHBG, per 5 nmol/L for IGF-1, per 400 pmol/L increment for total oestradiol and per 5 pmol/L increment for calculated free oestradiol); the increments were chosen based on the standard deviations of hormone concentrations. HRs and 95% CIs for the quintiles of hormone concentrations are also presented for comparison with the results from a large international consortium.^5–8^

The multivariable models were adjusted for ethnicity (White, others, unknown—0.3%), educational level (University degree or other professional qualification, A level or equivalent, O level or equivalent, other, unknown—1.0%), smoking (never, past, current, unknown—0.4%), alcohol use (3 times a month or less, 1–4 times a week, daily or almost daily, unknown—0.1%), physical activity quartiles (unknown—26.2%), body mass index (BMI) (<22.5 kg/m^2^, 22.5–24.9 kg/m^2^, 25–27.4 kg/m^2^, 27.5–29.9 kg/m^2^, 30–34.9 kg/m^2^, ≥35 kg/m^2^, unknown—0.3%), regular menstrual cycle (yes/no; pre-menopausal women only), parity and age at first birth (nulliparous, one— <25 years, 25–29 years, ≥30 years, two— <25 years, 25–29 years, ≥30 years, three or more— <25 years, 25–29 years, ≥30 years, unknown—0.3%), time since last oral contraceptive pill (OCP) use (never, 1–5 years, 6–10 years, 11–20 years and ≥21 years, unknown—8.7%), age at menopause (i.e. when periods stopped; <45 years, 45–49 years, 50–54 years, ≥55 years, unknown—12.9%; post-menopausal women only), time since hormone replacement therapy use (never, 1–5 years, 6–10 years and ≥11 years, unknown—5.7%; post-menopausal women only), presence of endocrine disorders (yes/no, unknown—0.02%) and family history of breast cancer in first-degree relatives (yes/no, unknown—1.4%). The models were also mutually adjusted for other hormones: associations with testosterone were adjusted for SHBG, IGF-1 and in pre-menopausal women total oestradiol; associations with IGF-1 were adjusted for total testosterone, SHBG and in pre-menopausal women total oestradiol; and associations with oestradiol were adjusted for total testosterone, SHBG and IGF-1.

The HRs and 95% CIs for total and calculated free testosterone, SHBG and IGF-1 were additionally corrected for regression dilution bias by assigning the median values from the quintiles of hormone concentrations measured in a subsample of women at the repeat assessment visit to the quintiles of hormone concentrations measured at recruitment.^10^ Women who reported currently using oral contraceptive pills or hormone replacement therapy at the repeat assessment visit, or who had a diagnosis of cancer prior to the visit were excluded from this subsample (*N* = 161). The HRs for total and calculated free oestradiol were not corrected as it was not possible to demonstrate correlations over time as mentioned above.

In pre-menopausal women, subgroup analyses were undertaken by age at blood collection (≤45 years vs. >45 years), by age at breast cancer diagnosis (≤50 years vs. >50 years) and by phase of the menstrual cycle. As time since the last menstrual period was assessed using the question “How many days since your last menstrual period?”, it was assumed that the days were counted from the last day of the last period and that the period on average lasted three days. Cycle phases were then defined by forward dating as early follicular = days 0–5, late follicular = days 6–10, mid-cycle = days 11–14, early luteal = days 15–18, mid-luteal = days 19–24 and late luteal = days ≥25.^6^ The HRs per 100 pmol/L of total oestradiol and per 1 pmol/L of calculated free oestradiol were presented for cycle phase-specific analyses.

In both pre- and post-menopausal women, sensitivity analyses were undertaken by excluding the first two years of follow-up (to assess reverse causality, i.e. the possible effects of preclinical tumours on hormone concentrations), and by excluding women with self-reported endocrine disorders, those with irregular menstrual cycles (pre-menopausal women only) at baseline and those with a concentration of testosterone or oestradiol (pre-menopausal women only) which was below the reportable range.

## Results

In total, 30,565 pre-menopausal and 133,294 post-menopausal women were included in the analyses, of whom 527 and 2997, respectively, experienced invasive breast cancer during a median follow-up of 7.1 years. Table 1 presents the characteristics of the study participants.

**Table 1:** Participant characteristics.

	Pre-menopausal women		Post-menopausal women	
	Cases (*N* = 527)	Non-cases (*N* = 30,038)	Cases (*N* = 2997)	Non-cases (*N* = 130,297)
Age at recruitment (years), mean (SD)	45.0 (2.6)	44.7 (2.7)	61.2 (4.9)	60.5 (5.3)
Townsend deprivation scores, median (IQR)	−1.9 (4.1)	−1.8 (4.5)	−2.3 (3.7)	−2.3 (3.9)
White, %	91.8	90.8	96.2	95.5
College or university degree	53.7	48.4	38.0	38.9
Current smoker, %	9.7	11.0	7.9	8.0
One or more drinks weekly, %	67.0	63.8	63.1	61.5
Self-reported physical activity (MET-h/week), median (IQR)	24.6 (36.5)	29.0 (39.8)	27.8 (41.3)	30.2 (45.6)
Body mass index (kg/m^2^), mean (SD)	26.0 (4.8)	26.3 (5.3)	28.0 (5.1)	27.3 (5.1)
Regular menstrual cycle, %	87.9	87.9		
Nulliparous, %	33.0	26.7	15.1	15.4
Age at first birth (years), mean (SD)	28.7 (5.6)	27.8 (5.5)	25.7 (5.0)	25.4 (4.8)
Past use of oral contraceptive, %	87.1	87.5	76.0	76.8
Time since oral contraceptive use (years), mean (SD)	13.8 (7.3)	14.5 (7.0)	28.0 (8.1)	27.4 (8.4)
Age at menopause, i.e. when periods stopped (years), mean (SD)			50.1 (5.6)	49.5 (5.7)
Past use of hormone replacement therapy, %			48.9	46.0
Time since hormone replacement therapy use (years), mean (SD)			7.6 (5.3)	7.7 (5.1)
Presence of endocrine disorders, %	4.7	6.7	15.0	14.0
Family history of breast cancer, %	8.4	5.7	10.2	6.7
Total testosterone (nmol/L), median (IQR)	1.12 (0.75)	1.12 (0.71)	0.94 (0.75)	0.85 (0.74)
Total testosterone—coefficient of variation, %	54.1	54.4	62.0	71.7
Calculated free testosterone (pmol/L), median (IQR)	12.69 (10.33)	12.49 (10.32)	12.29 (11.35)	10.46 (10.63)
Calculated free testosterone—coefficient of variation, %	67.8	70.3	71.4	85.0
SHBG (nmol/L), median (IQR)	62.60 (36.06)	62.95 (38.24)	50.79 (30.38)	53.58 (33.64)
SHBG—coefficient of variation, %	43.8	44.9	45.6	45.9
IGF-1 (nmol/L), median (IQR)	23.80 (6.81)	23.34 (6.86)	20.21 (7.06)	19.96 (7.13)
IGF-1—coefficient of variation, %	22.1	22.9	26.6	26.8
Total oestradiol (pmol/L), median (IQR)	335.7 (351.6)	347.8 (329.5)		
Total oestradiol—coefficient of variation, %	89.9	86.0		
Calculated free oestradiol (pmol/L), median (IQR)	3.89 (4.25)	4.10 (3.89)		
Calculated free oestradiol—coefficient of variation, %	83.2	79.8		

*SD* standard deviation, *IQR* interquartile range, *Met* metabolic equivalent of task, *SHBG* sex hormone-binding globulin, *IGF-1* insulin-like growth factor-1.

### Total and calculated free testosterone

**Fig. 1:**
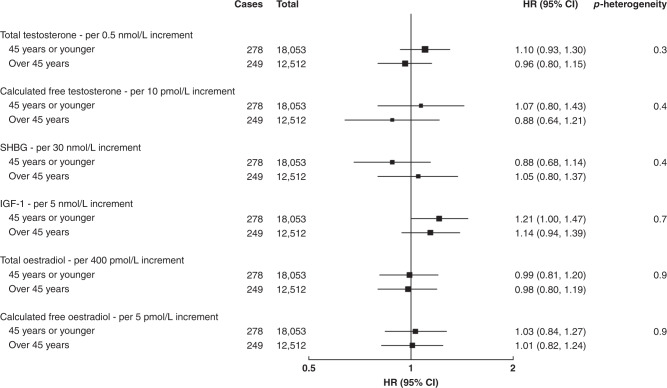
Associations of hormones and SHBG with the risk of invasive breast cancer by age at blood collection in pre-menopausal women. Hazard ratios stratified for age group, region and deprivation; adjusted for age (underlying time variable), ethnicity, educational level, smoking, alcohol, physical activity, BMI, regular menstrual cycle, parity, age at first birth, time since OCP use, presence of endocrine disorders, family history of breast cancer and other hormones and SHBG; and corrected for regression dilution using repeat measures except for total and calculated free oestradiol.

**Fig. 2:**
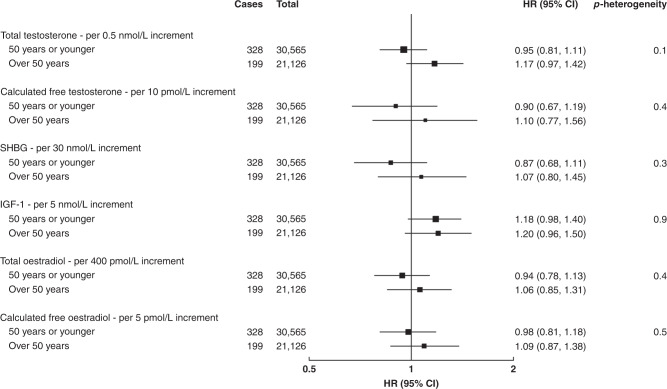
Associations of hormones and SHBG with the risk of invasive breast cancer by age at breast cancer diagnosis in pre-menopausal women. Hazard ratios stratified for age group, region and deprivation; adjusted for age (underlying time variable), ethnicity, educational level, smoking, alcohol, physical activity, BMI, regular menstrual cycle, parity, age at first birth, time since OCP use, presence of endocrine disorders, family history of breast cancer and other hormones and SHBG; and corrected for regression dilution using repeat measures except for total and calculated free oestradiol.

In pre-menopausal women, there were no significant associations between total and calculated free testosterone and breast cancer risk overall (Table 2 and Supplementary Fig. S2), or in subgroup analyses by age at blood collection (Fig. 1), by age at breast cancer diagnosis (Fig. 2) or by phase of the menstrual cycle (Supplementary Table S1).

**Table 2:** Associations of hormones and SHBG with the risk of invasive breast cancer in pre- and post-menopausal women.

	Cases	Total	HR (95% CI)^a^	HR (95% CI)^b^	HR (95% CI)^c^	HR (95% CI)^d^	HR (95% CI)^e^	*p*_heterogeneity_
Total testosterone, per 0.5 nmol/L increment								
Pre-menopausal	527	30,565	1.02 (0.94, 1.12)	1.02 (0.93, 1.12)	1.03 (0.94, 1.12)	1.01 (0.92, 1.11)	1.03 (0.92, 1.16)	0.03
Post-menopausal	2997	133,294	1.19 (1.14, 1.23)	1.19 (1.14, 1.23)	1.17 (1.12, 1.21)	1.16 (1.12, 1.21)	1.18 (1.14, 1.23)	
Calculated free testosterone, per 10 pmol/L increment								
Pre-menopausal	527	30,565	1.00 (0.89, 1.13)	1.01 (0.89, 1.13)	1.02 (0.90, 1.16)	0.97 (0.84, 1.12)	0.97 (0.78, 1.20)	0.009
Post-menopausal	2997	133,294	1.32 (1.26, 1.39)	1.32 (1.26, 1.39)	1.27 (1.20, 1.33)	1.25 (1.18, 1.32)	1.31 (1.23, 1.40)	
SHBG, per 30 nmol/L increment								
Pre-menopausal	527	30,565	0.98 (0.88, 1.08)	0.96 (0.87, 1.07)	0.94 (0.84, 1.06)	0.97 (0.86, 1.09)	0.96 (0.79, 1.15)	0.4
Post-menopausal	2997	133,294	0.83 (0.79, 0.87)	0.83 (0.79, 0.88)	0.89 (0.84, 0.94)	0.90 (0.85, 0.95)	0.89 (0.84, 0.94)	
IGF-1, per 5 nmol/L increment								
Pre-menopausal	527	30,565	1.14 (1.03, 1.25)	1.13 (1.03, 1.24)	1.13 (1.02, 1.24)	1.12 (1.02, 1.24)	1.18 (1.03, 1.36)	0.2
Post-menopausal	2997	133,294	1.07 (1.02, 1.11)	1.06 (1.02, 1.11)	1.08 (1.04, 1.12)	1.05 (1.01, 1.10)	1.07 (1.01, 1.12)	
Total oestradiol, per 400 pmol/L increment								
Pre-menopausal	527	30,565	1.00 (0.88, 1.14)	0.99 (0.86, 1.13)	0.98 (0.86, 1.13)	0.99 (0.86, 1.13)	–	–
Calculated free oestradiol, per 5 pmol/L increment								
Pre-menopausal	527	30,565	1.04 (0.90, 1.20)	1.03 (0.89, 1.19)	1.04 (0.90, 1.20)	1.03 (0.89, 1.19)	–	–

^a^Hazard ratios stratified for age group, region and deprivation and adjusted for age (underlying time variable).

^b^As above + adjusted for ethnicity, educational level, smoking, alcohol, physical activity, regular menstrual cycle (pre-menopause), parity, age at first birth, time since OCP use, age at menopause (post-menopause), time since HRT use (post-menopause), presence of endocrine disorders and family history of breast cancer.

^c^As above + adjusted for BMI.

^d^As above + adjusted for other hormones and SHBG.

^e^As above + corrected for regression dilution using repeat measures except for total and calculated free oestradiol.

In post-menopausal women, there were positive associations between total and calculated free testosterone and breast cancer risk (HR per 0.5 nmol/L increment: 1.18; 95% CI: 1.14, 1.23 and HR per 10 pmol/L increment: 1.31; 95% CI: 1.23, 1.40, respectively) (Table 2 and Supplementary Fig. S2).

There was a significant difference in the HRs per absolute increment of total and calculated free testosterone between pre- and post-menopausal women (*p*_heterogeneity_ = 0.03 for total testosterone and 0.009 for calculated free testosterone) (Table 2).

### SHBG

In pre-menopausal women, there was no significant association between SHBG concentration and breast cancer risk in all analyses (Table 2, Figs. 1, 2 and Supplementary Table S1, Supplementary Fig. S2).

In post-menopausal women, there was a significant inverse association between SHBG and breast cancer risk (HR per 30 nmol/L increment: 0.89; 95% CI: 0.84, 0.94) (Table 2 and Supplementary Fig. S2).

There was no significant difference in the associations between pre- and post-menopausal women (Table 2).

### IGF-1

In pre-menopausal women, there was a significant positive association between IGF-1 concentration and breast cancer risk (HR per 5 nmol/L increment: 1.18; 95% CI: 1.02, 1.35) (Table 2 and Supplementary Fig. S2). The association did not differ significantly by age at blood collection (Fig. 1), by age at breast cancer diagnosis (Fig. 2), or by phase of the menstrual cycle (Supplementary Table S1).

In post-menopausal women, there was a weaker positive association between IGF-1 and breast cancer risk (HR per 5 nmol/L increment: 1.07; 95% CI: 1.01, 1.12) (Table 2 and Supplementary Fig. S2). The apparent difference in the associations between pre- and post-menopausal women was not statistically significant (Table 2).

### Total and calculated free oestradiol in pre-menopausal women only

**Fig. 3:**
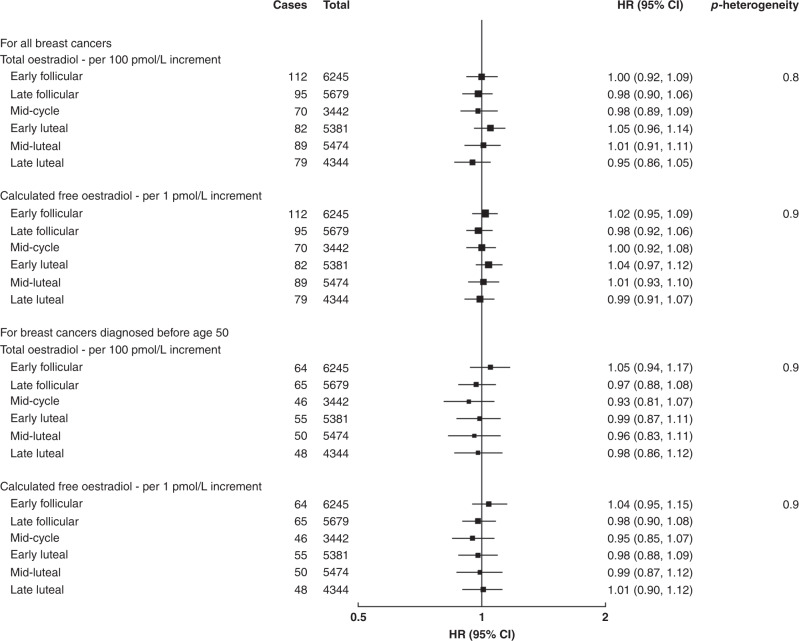
Associations of total and calculated free oestradiol with the risk of invasive breast cancer by phase of the menstrual cycle in pre-menopausal women. Hazard ratios stratified for age group, region and deprivation; and adjusted for age (underlying time variable), ethnicity, educational level, smoking, alcohol, physical activity, BMI, regular menstrual cycle, parity, age at first birth, time since OCP use, presence of endocrine disorders, family history of breast cancer and other hormones and SHBG.

**Fig. 4:**
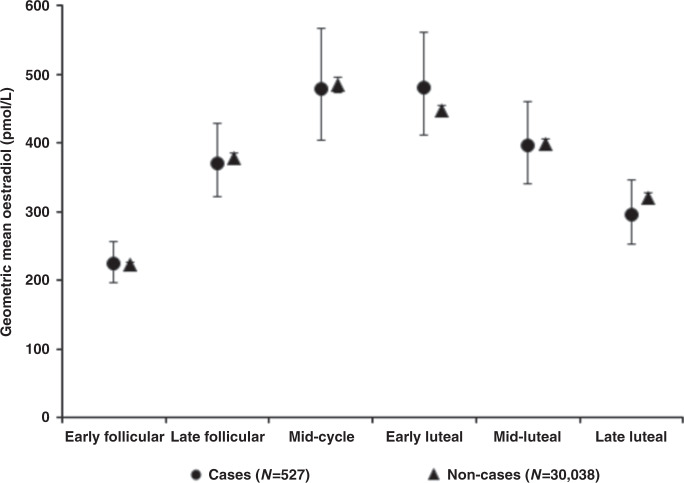
Geometric mean concentrations of total oestradiol by phase of the menstrual cycle in pre-menopausal women. Menstrual cycle phases were defined by forward dating as early follicular = days 0–5, late follicular = days 6–10, mid-cycle = days 11–14, early luteal = days 15–18, mid-luteal = days 19–24 and late luteal = days ≥25.

There was no significant association between total or calculated free oestradiol, standardised for the day of the menstrual cycle at blood collection, and breast cancer risk in pre-menopausal women overall (Table 2 and Supplementary Fig. S2), or in subgroup analyses by age at blood collection (Fig. 1), by age at breast cancer diagnosis (Fig. 2), or by phase of the menstrual cycle (Fig. 3). The geometric mean concentrations of total oestradiol across different phases of the menstrual cycle are shown in Fig. 4.

### Sensitivity analyses

Similar associations in relation to sex hormones and SHBG were observed after excluding the first two years of follow-up (Supplementary Fig. S3), and after excluding pre- and post-menopausal women with self-reported endocrine disorders, pre-menopausal women with irregular menstrual cycles, pre- and post-menopausal women with a concentration of testosterone which was below the reportable range, and pre-menopausal women with a concentration of oestradiol below the reportable range (Supplementary Table S2).

### Comparisons with the results from an international consortium

We compared our findings with those from a large international consortium of prospective studies.^5–8^ Pre-menopausal women in the consortium were on average 5 years younger than in the UK Biobank. There was also substantial between-laboratory variation in hormone measurement across studies. The consortium reported significant associations of breast cancer risk with total testosterone, SHBG, IGF-1 and total and calculated free oestradiol in pre-menopausal women and with total testosterone, SHBG and IGF-1, as well as with total and calculated free oestradiol, in post-menopausal women. The quintile-specific associations were not significantly different from the UK Biobank (Supplementary Fig. S2), except that the association with IGF-1 in pre-menopausal women was significantly weaker in the consortium than in the UK Biobank (*p*_heterogeneity in trends_ = 0.03) and the association with total testosterone in post-menopausal women was significantly stronger in the consortium (*p*_heterogeneity in trends_ = 0.02).

## Discussion

In this study, involving about 160,000 women, there were significant associations of testosterone, IGF-1 and SHBG with the risk of invasive breast cancer, with evidence of heterogeneity observed by menopausal status for testosterone.

### Total and calculated free testosterone

The relationships of testosterone (and other androgens) with the development of breast cancer are not well understood; testosterone can be converted to oestradiol by aromatase in adipose tissue and other peripheral tissues including breast tumour cells, and might also have direct effects through the androgen receptor which is commonly present in breast cancer cells.^14^ We found a moderately large positive association between total and calculated free testosterone and breast cancer risk in post-menopausal women but not in pre-menopausal women. In the collaborative reanalyses undertaken by the consortium, the associations of risk with total and free testosterone were also larger in post- than in pre-menopausal women, and the association with free testosterone was not significant in pre-menopausal women (Supplementary Fig. S2).^5–7^ The reason for this differential association by menopausal status is not known but might be related to the role of circulating testosterone in post-menopausal women as a substrate for the peripheral synthesis of oestradiol by aromatisation, or other factors. We were not able to account for concentrations of oestradiol in assessing the effect of testosterone in post-menopausal women as suggested previously^15^ although we did so for pre-menopausal women.

### SHBG

SHBG is a serum glycoprotein that binds oestrogens and androgens with high affinity and specificity,^16^ thereby lowering the bioavailable fraction of these sex steroids and potentially reducing subsequent breast cancer risk. We found an inverse association between SHBG and breast cancer risk in post-menopausal women but no apparent association in pre-menopausal women, similar to the results reported in the collaborative reanalyses (Supplementary Fig. S2).^6,7^

### IGF-1

IGF-1 is a polypeptide that stimulates growth. It may increase the risk of breast cancer by stimulating cell proliferation and inhibiting apoptosis.^17^ The effects of IGF-1 on breast cells may be enhanced by oestrogens^18^ as it has been proposed that IGF-1 interacts with oestrogen signalling pathways to regulate mitosis, apoptosis, adhesion, migration and differentiation of mammary epithelial cells.^19^ The overall association between IGF-1 and breast cancer risk in the UK Biobank cohort has been reported in a recent paper;^20^ in the current paper, we presented more detailed results separately for pre- and post-menopausal women; we found a positive association between IGF-1 and breast cancer risk in both pre- and post-menopausal women, as observed in the collaborative re-analysis of individual participant data from 17 prospective studies (Supplementary Fig. S2).^8^ Similar associations also apply to women with a family history of breast cancer.^21^

### Total and calculated free oestradiol in pre-menopausal women

Endogenous oestrogens may increase the risk of breast cancer by promoting cell proliferation and decreasing apoptosis.^1^ Higher oestradiol concentrations have been consistently associated with an increased risk of post-menopausal breast cancer.^5,7,22^ However, the evidence in pre-menopausal women is more limited due to fewer data being available and the complexity of conducting studies due to the cyclic nature of hormone concentrations as well as the cyclic nature of mammary cell proliferation.^23^ In the collaborative re-analysis of individual participant data from seven prospective studies there was a significant positive association of oestradiol (adjusted for cycle phase at blood collection) with breast cancer risk (Supplementary Fig. S2).^6^ However, we found no overall association of oestradiol (adjusted for cycle day) with risk. This should not be interpreted as a clear null finding because of certain limitations in the data for oestradiol, due both to the type of assay used and to the data available on the menstrual cycle phase.

The chemiluminescent immunoassays used in the UK Biobank were not as sensitive as more specialised assays for oestradiol^24^ and were not able to detect concentrations lower than 175 pmol/L, which is higher than the median concentration of 150 pmol/L expected in the early follicular phase;^25^ and consequently the test results were too low to be recorded by the assay in 20% of pre-menopausal women. We included these women in our analyses by setting the concentration at three-quarters of the minimum reportable value, but it is likely that this limitation reduces statistical power.

We did not find any significant difference in the association by cycle phase, whereas the association was significant in the follicular phase and at mid-cycle in the collaborative re-analysis.^6^ This apparent discrepancy in the results might be related to differences in the characterisation of the menstrual cycle phase in the two studies, as reflected in a much clearer variation of oestradiol with the cycle phase in the collaborative group than in the current analysis (shown in Fig. 4). Ideally, the phase of the menstrual cycle in observational epidemiological studies should be categorised by ‘backward dating’, i.e. counting the number of days backwards from the start of the subsequent menstrual period to the date of the blood sample, because most of the variation in the cycle length is in the follicular rather than the luteal phase.^26^ In the UK Biobank, the question used to assess time since the last menstrual period was ambiguous, and it was not possible to use backward dating to define cycle phase because the date of the menstrual period following blood collection was not recorded, whereas in the majority of studies included in the collaborative re-analysis more specific questions were used and backward dating was possible. Therefore, the information that UK Biobank provides on oestradiol in pre-menopausal women has limitations.

### Effects of potential confounders

To minimise residual confounding, we included all the measured factors (demographic, lifestyle, anthropometric and reproductive) known to be associated with breast cancer risk in the multivariable-adjusted models. Many of these factors including age, education, smoking, alcohol consumption and body mass index have also been associated with hormone concentrations although the associations of hormones with other factors such as parity and age at first birth appear to be modest.^27–29^ However, adjustment for all these factors including body mass index had minimal impact on the associations of hormones and SHBG with breast cancer risk (as shown in Table 2). Moreover, mutual adjustment for hormones did not substantially affect the associations.

### Causality of observed associations and implications

The likelihood that the associations observed in this analysis are causal is supported by findings from some previous Mendelian randomisation (MR) studies and randomised controlled trials (RCTs). Recent MR studies have shown that genetically predicted testosterone and IGF-1 are both associated with a higher risk of breast cancer,^20,30^ and that genetically predicted SHBG is associated with a lower risk of breast cancer;^31^ it should be noted, however, that these genetic associations may not demonstrate direct causal effects, since testosterone might affect risk by acting as a precursor of oestradiol, and SHBG probably affects risk by regulating the bioavailability of sex hormones. Our finding of a significantly greater risk associated with increased testosterone in post- compared with pre-menopausal women could be viewed as support for an indirect rather than a direct effect of this hormone because oestradiol is synthesised from testosterone in peripheral tissues through the action of aromatase in post-menopausal women. Although we did not find a significant association of breast cancer risk with oestradiol in pre-menopausal women, and we are not aware of evidence from MR studies, previous RCTs have shown that the selective oestrogen receptor modulator tamoxifen, which reduces the activation of the oestrogen receptor by oestradiol, reduces risk in pre-menopausal women.^32,33^

Given the complex interplay between sex hormones and growth factors, potential genetic pleiotropy in the MR studies, and potential differential effects of hormones by menopausal status as observed in this analysis, more research is needed to better understand the causal role of hormones and SHBG in breast carcinogenesis. Nevertheless, our findings may help inform current efforts to identify women at comparatively high risk, who would benefit from more intensified screening, chemoprevention with hormonal therapy or other risk-reducing measures. The performance of the existing risk prediction models has been suboptimal,^34^ but recent research found that incorporation of hormonal biomarkers improved the models’ discrimination particularly in post-menopausal women.^35–37^

### Strengths and limitations

The main strength of our study is a combination of the prospective design, a very large sample size with hormones measured for the full cohort, nearly complete (98–99%) case ascertainment by cancer registries^38^ and the ability to account for regression dilution bias using a large repeat sample with about 5000 women.

The limitations of the information on oestradiol and the phase of the menstrual cycle have been discussed above. A further limitation is for the testosterone assay, for which 16% of women had values below the lower limit of detection; this is likely to have reduced statistical power and the strength of the association, but would not be expected to otherwise bias the results. Other limitations are the lack of data on other hormones such as progesterone and androgens other than testosterone, lack of information on the hormone receptor status of the breast cancer cases, and the study sample comprising predominantly white women aged over 40 years.

## Summary and conclusions

In summary, we found a positive association of breast cancer risk with testosterone in post-menopausal women; an inverse association of risk with SHBG in post-menopausal women; and a positive association of risk with IGF-1 in both pre- and post-menopausal women. We did not find a significant association with oestradiol, possibly due to the technical limitations discussed above. Overall, the findings from this large prospective cohort study confirm significant associations of testosterone, IGF-1 and SHBG with the risk of invasive breast cancer, with evidence of heterogeneity by menopausal status for testosterone.

## Supplementary information

Supplementary tables and figures

## Data Availability

UK Biobank is an open-access resource, and the study website https://www.ukbiobank.ac.uk/ has information on available data and access procedures.
