# Supplementary material for: Endogenous hormones and risk of invasive breast cancer in pre- and post-menopausal women: findings from the UK Biobank
Source: Br J Cancer. 2021 Apr 16;125(1):126–34. doi: 10.1038/s41416-021-01392-z (PMC8257641; doi:10.1038/s41416-021-01392-z)
Supplement: Supplementary file 1 — Supplementary tables and figures [file 41416_2021_1392_MOESM1_ESM.docx]

**Table S1. Associations of hormones and SHBG with the risk of invasive breast cancer by phase of menstrual cycle in pre-menopausal women**

|  | **HR (95% CI)** | | | | | | **p-heterogeneity** |
| --- | --- | --- | --- | --- | --- | --- | --- |
|  | **Early follicular** | **Late follicular** | **Mid-cycle** | **Early luteal** | **Mid-luteal** | **Late luteal** |  |
|  | 6,245 women; 112 cases | 5,679 women; 95 cases | 3,442 women; 70 cases | 5,381 women; 82 cases | 5,474 women; 89 cases | 4,344 women; 79 cases |  |
| Total testosterone per 0.5 nmol/L increment | 1.02 (0.79, 1.33) | 1.06 (0.79, 1.42) | 1.08 (0.73, 1.60) | 1.04 (0.75, 1.45) | 1.06 (0.78, 1.46) | 0.92 (0.65, 1.30) | 0.9 |
| Calculated free testosterone per 10 pmol/L increment | 0.99 (0.63, 1.58) | 0.93 (0.55, 1.58) | 0.96 (0.48, 1.89) | 1.08 (0.61, 1.92) | 1.07 (0.60, 1.89) | 0.74 (0.41, 1.33) | 0.9 |
| SHBG per 30 nmol/L increment | 0.98 (0.64, 1.50) | 0.77 (0.48, 1.23) | 1.11 (0.63, 1.94) | 0.80 (0.49, 1.30) | 0.88 (0.55, 1.39) | 1.04 (0.61, 1.77) | 0.9 |
| IGF-1 per 5 nmol/L increment | 1.13 (0.82, 1.56) | 1.26 (0.89, 1.77) | 1.31 (0.85, 2.01) | 1.03 (0.71, 1.51) | 1.31 (0.90, 1.90) | 1.17 (0.79, 1.74) | 0.9 |

Hazard ratios stratified for age group, region and deprivation; adjusted for age (underlying time variable), ethnicity, educational level, smoking, alcohol, physical activity, BMI, regular menstrual cycle, parity, age at first birth, time since OCP use, presence of endocrine disorders, family history of breast cancer and other hormones and SHBG; and corrected for regression dilution using repeat measures.

**Table S2. Sensitivity analyses**

|  | **HR (95% CI)** | | | |
| --- | --- | --- | --- | --- |
|  | **Excluded women with self-reported endocrine disorders** | **Excluded women with irregular cycle** | **Excluded women with a concentration of testosterone below the reportable range** | **Excluded women with a concentration of oestradiol below the reportable range** |
| **Pre-menopausal women** | 28,529 women; 502 cases | 26,864 women; 463 cases | 28,631 women; 498 cases | 24,307 women; 407 cases |
| Total testosterone, per 0.5 nmol/L increment | 1.05 (0.93, 1.19) | 1.05 (0.92, 1.19) | 1.03 (0.90, 1.18) |  |
| Calculated free testosterone, per 10 pmol/L increment | 1.03 (0.83, 1.29) | 1.02 (0.81, 1.28) | 0.92 (0.73, 1.16) |  |
| SHBG, per 30 nmol/L increment | 0.94 (0.77, 1.13) | 0.96 (0.78, 1.17) |  |  |
| IGF-1, per 5 nmol/L increment | 1.19 (1.04, 1.38) | 1.19 (1.03, 1.38) |  |  |
| Total oestradiol, per 400 pmol/L increment | 0.97 (0.84, 1.11) | 1.01 (0.87, 1.17) |  | 1.07 (0.92, 1.24) |
| Calculated free oestradiol, per 5 pmol/L increment | 1.01 (0.87, 1.17) | 1.06 (0.91, 1.25) |  | 1.08 (0.92, 1.27) |
| **Post-menopausal women** | 114,561 women; 2,548 cases |  | 109,800 women; 2,602 cases |  |
| Total testosterone, per 0.5 nmol/L increment | 1.17 (1.12, 1.22) |  | 1.18 (1.12, 1.24) |  |
| Calculated free testosterone, per 10 pmol/L increment | 1.27 (1.19, 1.37) |  | 1.25 (1.15, 1.35) |  |
| SHBG, per 30 nmol/L increment | 0.89 (0.83, 0.95) |  |  |  |
| IGF-1, per 5 nmol/L increment | 1.07 (1.02, 1.14) |  |  |  |

Hazard ratios stratified for age group, region and deprivation; adjusted for age (underlying time variable), ethnicity, educational level, smoking, alcohol, physical activity, BMI, regular menstrual cycle (pre-menopause), parity, age at first birth, time since OCP use, age at menopause (post-menopause), time since HRT use (post-menopause), presence of endocrine disorders, family history of breast cancer and other hormones and SHBG; and corrected for regression dilution using repeat measures except for total and calculated free oestradiol.

**Figure S1. Flow diagram of study participants**

Total women (N=273,383)

Withdrawal (N=203)

**Post-menopausal women included (N=133,294)**

**Pre-menopausal women included (N=30,565)**

Missing data on recruitment date (N=11)

Prior diagnosis of cancer, benign CNS tumour, breast carcinoma in situ (N=15,660)

Taking HRT or use unknown (N=18,945)

Missing data on serum hormones and SHBG (N=26,086)

Pre-menopausal women (N=50,794)

Prior diagnosis of cancer, benign CNS tumour, breast carcinoma in situ (N=784)

Taking OCP or use unknown (N=5,606)

Missing data on serum hormones and SHBG (N=8,926)

Missing data on time since last menstrual period or >180 days since last menstrual period (N=4,452)

Being pregnant (N=461)

Menopausal status unknown or uncertain (N=28,390)

Post-menopausal women (N=193,996)

**Figure S2. Associations of hormones and SHBG with the risk of invasive breast cancer in pre- and post-menopausal women in UK Biobank vs. collaborative re-analyses**


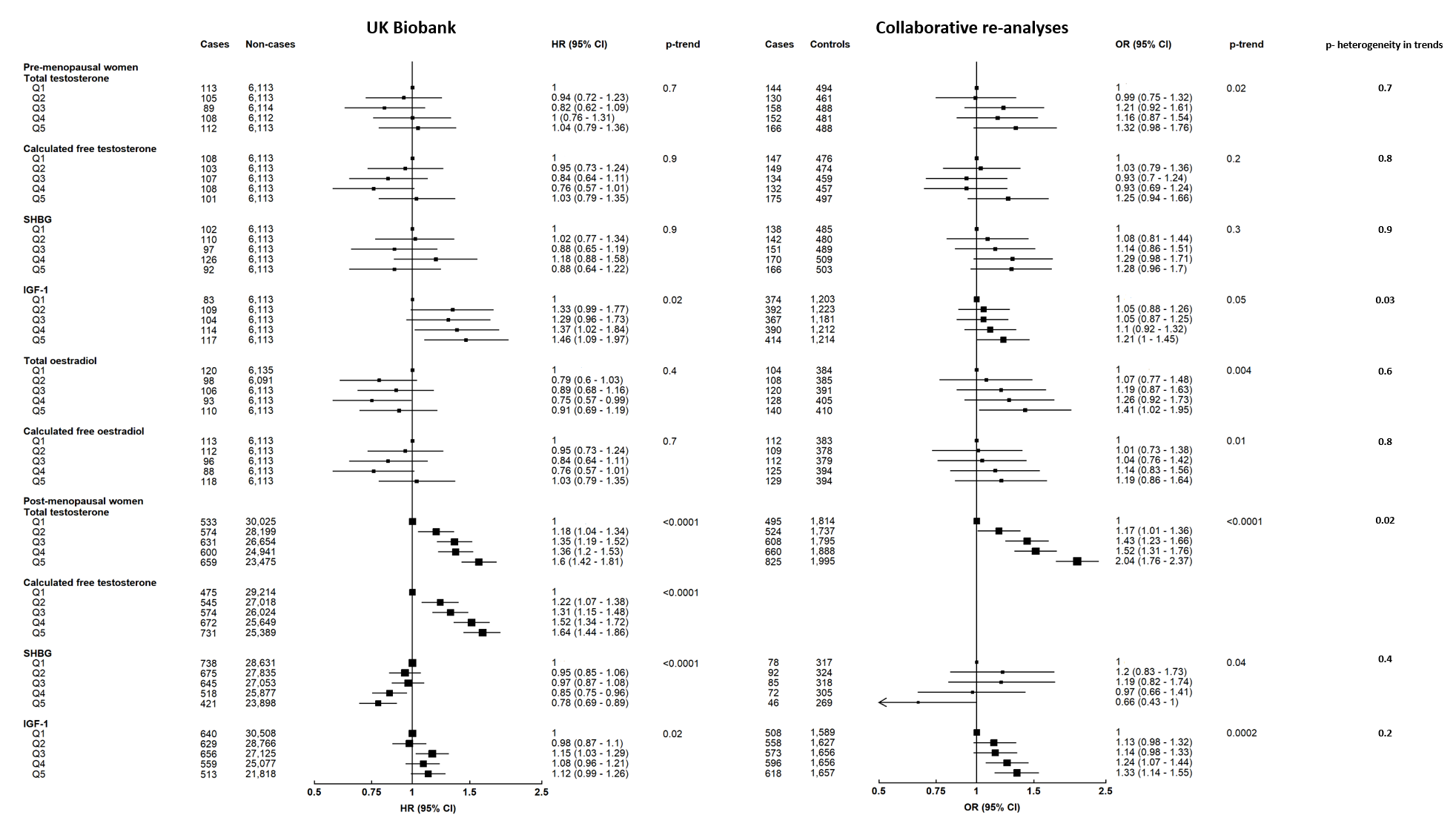


UK Biobank:

Hazard ratios stratified for age group, region and deprivation; adjusted for age (underlying time variable), ethnicity, educational level, smoking, alcohol, physical activity, BMI, regular menstrual cycle (pre-menopause), parity, age at first birth, time since OCP use, age at menopause (post-menopause), time since HRT use (post-menopause), presence of endocrine disorders, family history of breast cancer and other hormones and SHBG

Collaborative re-analyses:

Pre-menopausal women: Odds ratios matched on age and date of blood sample and on the day or phase of menstrual cycle at blood collection within each study^6^

Post-menopausal women: Odds ratios for total testosterone matched on age and date of blood sample within each study^5^; odds ratios for SHBG matched on age and date of blood sample within each study^7^; and odds ratios for IGF-1 matched by study, date of blood collection and age at blood collection^8^

**Figure S3. Associations of hormones and SHBG with the risk of invasive breast cancer after excluding the first two years of follow-up in pre- and post-menopausal women**

**
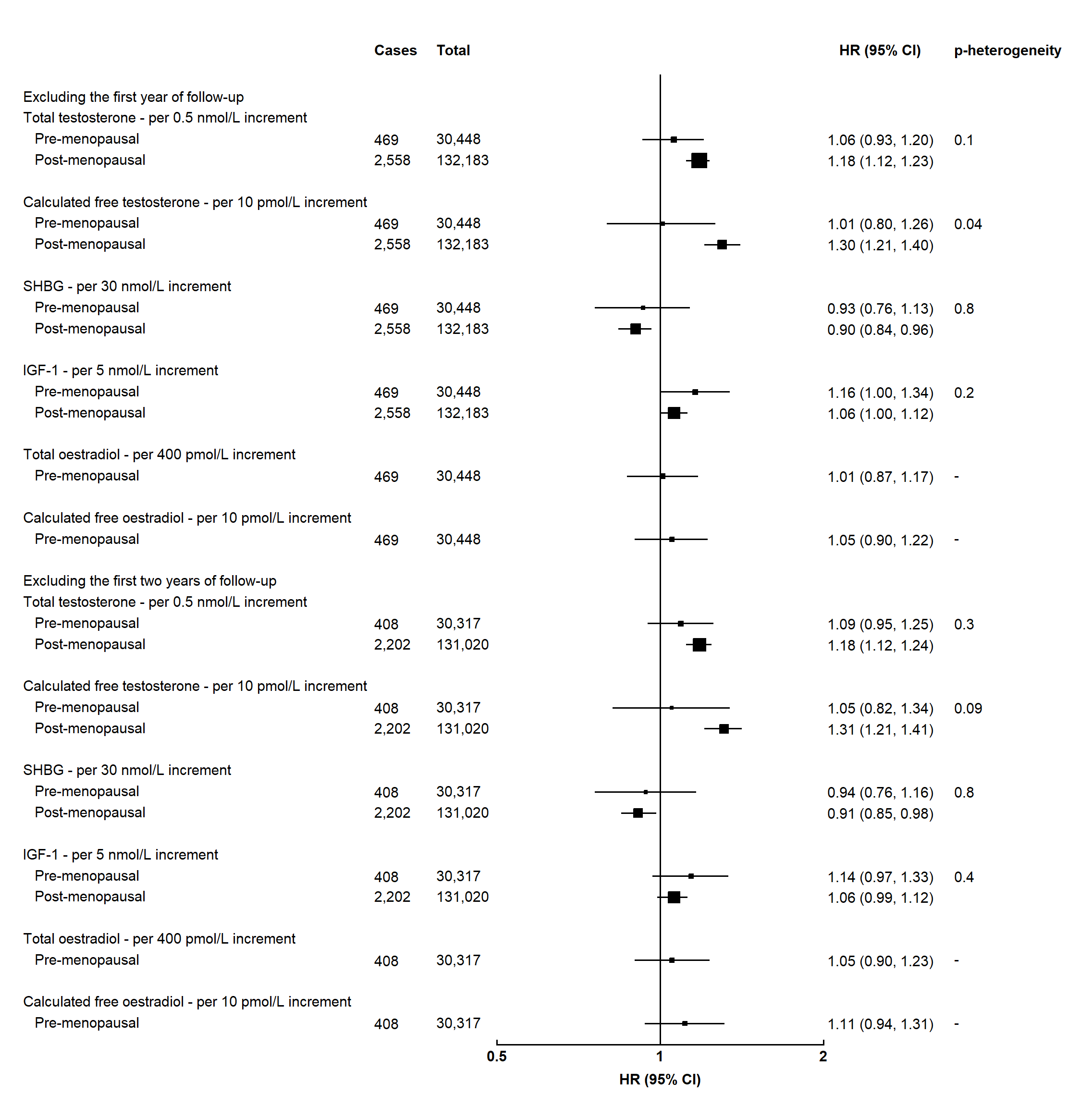
**

Hazard ratios stratified for age group, region and deprivation; adjusted for age (underlying time variable), ethnicity, educational level, smoking, alcohol, physical activity, diet, BMI, regular menstrual cycle (pre-menopause), parity, age at first birth, time since OCP use, age at menopause (post-menopause), time since HRT use (post-menopause), presence of benign breast lesions at baseline, presence of endocrine disorders, family history of breast cancer and other hormones and SHBG; and corrected for regression dilution using repeat measures except for total and calculated free oestradiol.
